# Understanding terror and violence in the lives of children and adolescents

**DOI:** 10.3402/ejpt.v5.25121

**Published:** 2014-07-02

**Authors:** Grete Dyb, Miranda Olff

**Affiliations:** 1 Norwegian Centre for Violence and Traumatic Stress Studies, NKVTS, Norway and Institute of Clinical Medicine, Faculty of Medicine, University of Oslo, Norway; 2 Department of Psychiatry, Academic Medical Centre, University of Amsterdam & Arq Psychotrauma Expert Group, Diemen, the Netherlands

Millions of children each year are exposed to acute events that affect one individual or family at a time (e.g., car accidents, residential fire, street violence, sudden medical events) (Langeland & Olff, 2008). Less frequent, but with major impact, are terror attacks. Across the world, terrorist groups, single actor terrorists, and perpetrators of school shootings have attacked groups of children and youth in spaces thought to provide safety. Research performed after such attacks suggests that the prevalence of posttraumatic stress reactions among persons with high levels of exposure is substantial (Schwarz & Kowalski, 1991; Scrimin et al., 2006).

This issue of the *European Journal of Psychotraumatology* focuses on recent major shooting events targeting youth in Europe. In Finland two shootings took place; the first school shooting occurred in Jokela 2007 and the second in Kauhajoki 2008. After the 2007 school shooting in Finland, high levels of posttraumatic distress were reported by 27% of females and 7% of males, 4 months after the shooting (Suomalainen, Haravuori, Berg, Kiviruusu, & Marttunen, 2011). In this issue, Turunen presents how attachment may be associated with the recovery processes following these events (Turunen, Haravuori, Punamäki, Suomalainen, & Marttunen, 2014).

Recently, the July 22, 2011, Norway terrorist attack horrified the world. Survivors of the shootings at Utøya were interviewed 4–5 months after the attack, and 47% had developed either “full posttraumatic stress disorder (PTSD)” or “partial PTSD” (Dyb et al., 2013). The horror of the events also spread to communities around Norway. Thoresen, Aakvaag, Wentzel-Larsen, Dyb, and Hjemdal (2012) reported strong immediate emotional responses, particularly sadness and a feeling of unreality, in the general population in Norway in the aftermath of the events. While Thoresen et al. found that PTSD reactions were elevated in ethnic minorities, in this special issue Nordanger et al. (2014) suggest that terror may have a more destabilizing impact on victims who have experienced prior adversities, in relation to their peers in college.

Another important issue addressed in this issue is that in communities struck by terror attacks, health care providers immediately are challenged by the question of how to adequately respond. Under-treatment and unmet needs among survivors have been repeatedly documented (Brewin et al., 2010) and can cause unnecessary suffering in survivors. Unfortunately, the question of how to ultimately respond is not fully resolved (Bisson, 2014). There is still little research on the efficacy of very early interventions and what does exist is largely evidence-informed rather than evidence-based. However, doing nothing to support victims of acute traumatic events is not an attractive option.

In this special issue, Nancy Kassam-Adams’ paper presents a framework for thinking about the design, delivery, and evaluation of early interventions for children who have been exposed to different kinds of acute trauma (Kassam-Adams, 2014). She points at targets for early intervention and describes the next steps for research and practice.

Five early intervention principles have been proposed: promoting a sense of safety, calming, a sense of self- and community-efficacy, connectedness and hope (Dückers, 2013; Hobfoll et al., 2007). Many recent early responses to major traumatic events have been guided by these principles, but the designs of actual outreach programs are heterogeneous. In this issue, two different outreach programs are presented. The Finish model mainly focused on outreach organized and administered at one site (Turunen, Haravuori, Pihlajamäki, Marttunen, & Punamäki, 2014), while the outreach to survivors and families in Norway was based on local resources in survivors’ municipalities (Dyb, Jensen, Glad, Nygaard, & Thoresen, 2014). Both outreach programs were based on proactiveness and provided resources for a longer period of time.

In Norway, outreach to school children was provided through an initiative from educational authorities, and in this issue, Schultz, Langballe, and Raundalen (2014) point out the difficulties children might have in understanding what happened and the teachers’ role in explaining acts of terror and establishing a sense of safety.

There remain many challenges to be addressed in preventing PTSD and other difficulties related to acute traumatic events. For obvious reasons, research in these highly unexpected situations may be very challenging and systematic observations are difficult to conduct. It is highly relevant to focus on ethical issues, and targeting victims of trauma for multiple enquiries by researchers should be avoided. Immediately after the terror attack in Norway, a coordinating initiative for research groups was established to promote collaboration between researchers and to protect victims from additional strain (Refsdal, 2014).

This issue is a reflection of the “ISTSS global meeting” organized by the Norwegian Center for Violence and Traumatic Stress Studies (NKVTS, nkvts.no) and the International Society for Traumatic Stress Studies (ISTSS, www.istss.org), in close collaboration with its European counterpart ESTSS (www.estss.org).

*Grete Dyb*
Norwegian Centre for Violence and
Traumatic Stress Studies
NKVTS
Norway and Institute of Clinical Medicine
Faculty of Medicine, University of Oslo
Norway Email: grete.dyb@nkvts.unirand.no *Miranda Olff*
Department of Psychiatry, Academic Medical Centre
University of Amsterdam &
Arq Psychotrauma Expert Group
Diemen, the Netherlands
